# The *EGFR* mutation status affects the relative biological effectiveness of carbon-ion beams in non-small cell lung carcinoma cells

**DOI:** 10.1038/srep11305

**Published:** 2015-06-11

**Authors:** Napapat Amornwichet, Takahiro Oike, Atsushi Shibata, Chaitanya S. Nirodi, Hideaki Ogiwara, Haruhiko Makino, Yuka Kimura, Yuka Hirota, Mayu Isono, Yukari Yoshida, Tatsuya Ohno, Takashi Kohno, Takashi Nakano

**Affiliations:** 1 Department of Radiation Oncology, Gunma University Graduate School of Medicine, Maebashi, Gunma, Japan; 2 Department of Radiology, Chulalongkorn University, Pathumwan, Bangkok, Thailand; 3 Division of Genome Biology, National Cancer Center Research Institute, Chuo-ku, Tokyo, Japan; 4 Advanced Scientific Research Leaders Development Unit, Gunma University, Maebashi, Gunma, Japan; 5 Department of Oncologic Sciences, Mitchell Cancer Institute, Alabama, USA; 6 Tottori University Hospital Cancer Center, Yonago, Tottori, Japan; 7 Gunma University Heavy Ion Medical Center, Maebashi, Gunma, Japan

## Abstract

Carbon-ion radiotherapy (CIRT) holds promise to treat inoperable locally-advanced non-small cell lung carcinoma (NSCLC), a disease poorly controlled by standard chemoradiotherapy using X-rays. Since CIRT is an extremely limited medical resource, selection of NSCLC patients likely to benefit from it is important; however, biological predictors of response to CIRT are ill-defined. The present study investigated the association between the mutational status of *EGFR* and *KRAS*, driver genes frequently mutated in NSCLC, and the relative biological effectiveness (RBE) of carbon-ion beams over X-rays. The assessment of 15 NSCLC lines of different *EGFR*/*KRAS* mutational status and that of isogenic NSCLC lines expressing wild-type or mutant *EGFR* revealed that *EGFR-*mutant NSCLC cells, but not *KRAS-*mutant cells, show low RBE. This was attributable to (i) the high X-ray sensitivity of *EGFR*-mutant cells, since *EGFR* mutation is associated with a defect in non-homologous end joining, a major pathway for DNA double-strand break (DSB) repair, and (ii) the strong cell-killing effect of carbon-ion beams due to poor repair of carbon-ion beam-induced DSBs regardless of *EGFR* mutation status. These data highlight the potential of *EGFR* mutation status as a predictor of response to CIRT, i.e., CIRT may show a high therapeutic index in *EGFR* mutation-negative NSCLC.

Locally-advanced non-small cell lung carcinoma (NSCLC) has poor prognosis. The 5 year overall survival rate of standard chemoradiotherapy using X-rays is 15–20%^1^,^2^,^3^. The local recurrence rate of approximately 30% in this population highlights the necessity for increased treatment intensity at primary disease sites; however, the characteristics of X-ray dose distribution limits further dose escalation in tumors with keeping tolerance dose in the surrounding organs such as the lung^4^,^5^. Therefore, a treatment modality with a higher efficacy than X-rays in primary tumors is required in locally-advanced NSCLC.

Carbon-ion radiotherapy (CIRT) has been provoking interest as a highly intensive local therapy. Carbon-ion beams have advantages over X-rays: a superior dose distribution associated with the sharp penumbra and the Bragg peak, and a strong cell-killing effect^6^,^7^. In early NSCLC, CIRT demonstrates a 5 year local control rate of 90–95%^8^,^9^. Based on these promising results, a clinical trial on CIRT in inoperable locally-advanced NSCLC was launched at Gunma University in 2013 (protocol number: GUNMA1201). Nevertheless, CIRT is currently an extremely limited medical resource, with fewer than ten facilities in the world; this situation may not be substantially improved in the next few decades because of high costs. Therefore, the selection of locally-advanced NSCLC cases in which CIRT is beneficial is of great importance.

Recent genome-wide mutation analyses revealed that NSCLCs possess genetic alterations, called “driver gene mutations”, that play significant roles in carcinogenesis by abnormally activating oncogenes^10^,^11^,^12^. In most cases, these driver gene mutations are mutually exclusive^13^,^14^; in other words, NSCLCs can be classified based on driver gene mutation status. Drugs that target activated oncogene products have begun to replace conventional cytotoxic chemotherapy, even for first-line use^10^. However, little is known about the association between driver gene mutation status and relative biological effectiveness (RBE) of carbon-ion beams in NSCLC. If it affects RBE, the driver gene mutation status may be a useful predictor of response to CIRT. To investigate this issue, we analyzed the sensitivity of 15 NSCLC lines with known mutations in *EGFR* and *KRAS*, genes frequently mutated in NSCLC^13^,^14^, to X-rays and carbon-ion beams.

## Results and Discussion

**Figure 1:**
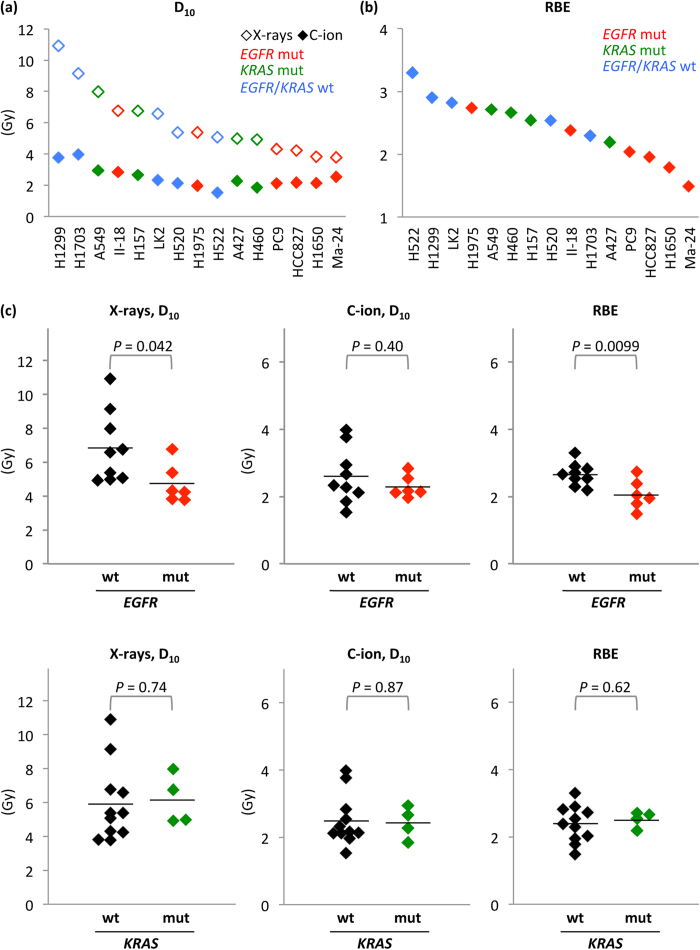
Sensitivity of *EGFR*-mutant, *KRAS*-mutant, or *EGFR/KRAS* wild-type NSCLC lines to X-rays or carbon-ion beams assessed by clonogenic survival assay. C-ion, carbon-ion; mut, mutant; wt, wild-type. The original survival curves are shown in Supplementary Figure 1. **(a)** D_10_ for X-rays and carbon-ion beams. **(b)** RBE of carbon-ion beams at D_10_. **(c)** Statistical analysis of the difference in D_10_ for X-rays or carbon-ion beams, or RBE of carbon-ion beams in NSCLC lines based on *EGFR* or *KRAS* mutation status. *P* values on the significant differences in the mean values (black lines) between mutant and wild-type lines were shown.

We first examined the sensitivity of 15 NSCLC lines with different *EGFR* and *KRAS* mutational statuses to X-rays or carbon-ion beams by clonogenic survival assay (Fig. 1a, Supplementary Fig. 1). The mutation status of these lines is listed in Supplementary Table 1^15^,^16^,^17^,^18^,^19^,^20^. The X-ray dose producing 10% survival (D_10_) varied widely among the cell lines (3.8–10.9). The D_10_ of *EGFR*-mutant lines was significantly lower than that of *EGFR* wild-type lines (Fig. 1c**, upper left panel**). These data indicate that NSCLC cells show varying degrees of sensitivity to X-rays and that *EGFR*-mutant cells are more sensitive to X-rays than *EGFR* wild-type cells. The D_10_ achieved by carbon-ion beams was lower than that achieved by X-rays in all of the cell lines and was scored within a relatively narrow range (1.5-4.0) (Fig. 1a). No significant difference was observed between the carbon-ion beam D_10_ in *EGFR*-mutant lines and that in *EGFR* wild-type lines (Fig. 1c**, upper middle panel**). These data indicate that carbon-ion beams have a higher cell-killing effect than X-rays regardless of *EGFR* mutation status. Carbon-ion beam RBEs calculated from the D_10_ values obtained for X-rays and carbon-ion beams ranged from 1.5 to 3.8 (Fig. 1b). The RBEs in *EGFR*-mutant lines were significantly lower than those in wild-type lines (Fig. 1c**, upper right panel**). In contrast to *EGFR*, there was no significant difference in X-ray D_10_, carbon-ion beam D_10_, and the RBE between the 4 *KRAS*-mutant and the 11 *KRAS* wild-type lines (Fig. 1a,b,c**, lower panels**).

**Figure 2:**
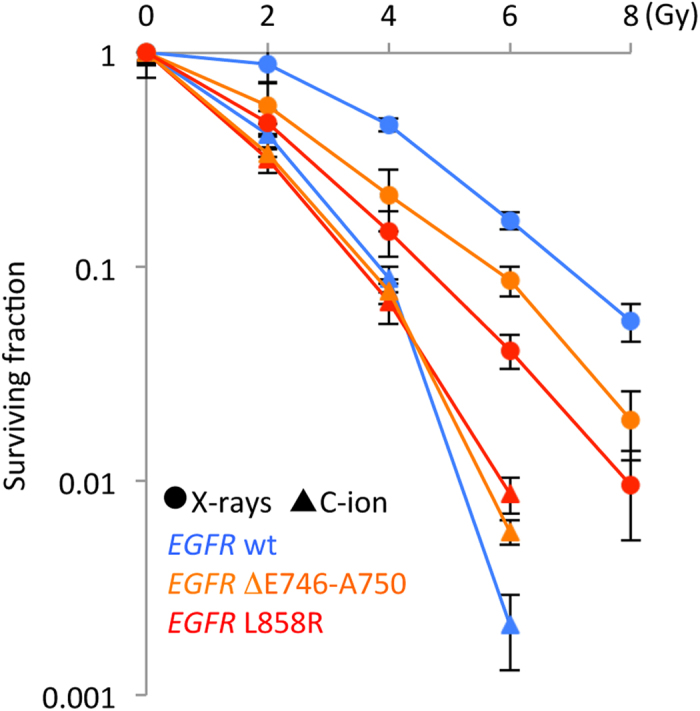
Sensitivity of isogenic A549 cells stably expressing wild-type or mutant (ΔE746-A750 or L858R) EGFR proteins to X-rays or carbon-ion beams assessed by clonogenic survival assay. Data are represented as mean ± standard deviations. C-ion, carbon-ion; wt, wild-type.

To confirm the greater X-ray sensitivity and RBE of *EGFR*-mutant cells compared to *EGFR* wild-type cells, we compared the sensitivity to X-rays and carbon-ion beams using isogenic A549 cells stably expressing wild-type or mutant EGFR proteins. A549-WT, A549-ΔE746-A750, and A549-L858R cells, which express, respectively, wild-type EGFR, a ΔE746-A750 deletion mutant, and a L858R point mutant, were used. Among the various *EGFR* mutations identified in human NSCLC, ΔE746-A750 and L858R are the most frequent (39.4%) and second most frequent (37.5%), respectively^21^. A549-ΔE746-A750 and A549-L858R cells showed higher sensitivity to X-rays than A549-WT cells (Fig. 2), but all three cell lines showed similar sensitivity to carbon-ion beams. Thus, the RBE observed in A549-ΔE746-A750 and A549-L858R cells was lower than that observed in A549-WT cells (Supplementary Table 2). Taken together, these data suggest that *EGFR*-mutant NCSLC cells show a low RBE due to their high sensitivity to X-rays and to the strong *EGFR* mutation-independent cell-killing effect of carbon-ion beams.

**Figure 3:**
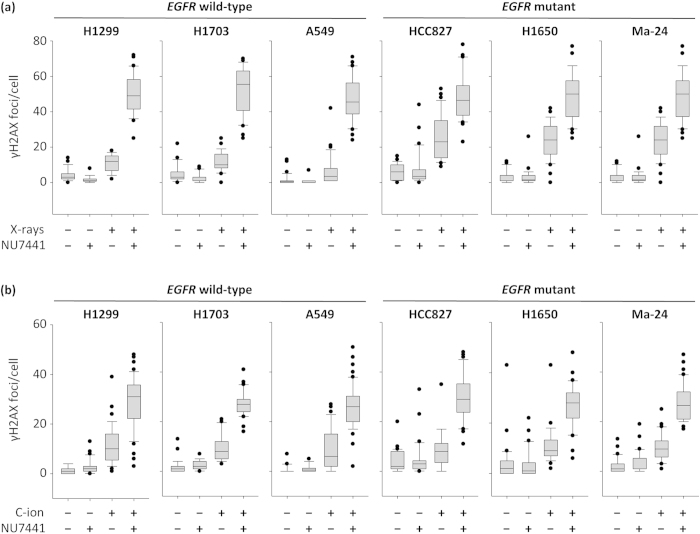
Repair of X-ray- or carbon-ion beam-induced DSBs in *EGFR*-mutant or wild-type NSCLC lines assessed by immunofluorescence staining of γH2AX. Cells were exposed to X-rays (6 Gy) or carbon-ion beams (2 Gy) in the presence or absence of the DNA-PKcs inhibitor NU7441 (10 μM) and stained with an antibody to γH2AX 24 h post-irradiation. The number of γH2AX foci per nucleus was scored in 30-50 cells for each experimental condition using a fluorescence microscope at ×100 magnification. The results of a representative experiment are shown as box plots. **(a)** X-rays. **(b)** Carbon-ion beams (C-ion).

DNA double-strand breaks (DSBs) are most critical lesions contributing to the cell-killing effect of ionizing irradiation. Therefore, we investigated the association between the *EGFR* mutation status and the capacity for repair of X-ray- or carbon-ion beam-induced DSBs. DSB repair was assessed by scoring the number of Ser139-phosphorylated histone H2AX (γH2AX) foci, which are markers for DSBs, 24 h post-irradiation using immunofluorescence staining^22^. H1299, H1703, and A549 cells, which showed the three highest D_10_ values for X-rays, and HCC827, H1650 and Ma-24 cells, which showed the three lowest, were used. In *EGFR* wild-type lines, the numbers of γH2AX foci after X-ray irradiation were slightly higher than those in non-treated controls, indicating that a major proportion of X-ray-induced DSBs were repaired 24 h post-irradiation (Fig. 3a). In *EGFR-*mutant lines, the numbers of γH2AX foci after X-ray irradiation were significantly higher than those in X-ray-irradiated *EGFR* wild-type lines (Fig. 3a, Supplementary Table 3). These data suggest that the capacity of *EGFR*-mutant cells for repair of X-ray-induced DSBs is lower than that of *EGFR* wild-type cells.

Previous studies showed that the EGFR ΔE746-A750 deletion mutant and L858R point mutant are defective in translocation to the nucleus and in binding to the catalytic subunit of DNA-dependent protein kinase (DNA-PKcs) in response to ionizing irradiation^23^,^24^,^25^. DNA-PKcs plays central roles in non-homologous end joining (NHEJ), a major DSB repair pathway^26^. After ionizing irradiation, DNA-PKcs is recruited to DSB sites and autophosphorylated. Then DNA-PKcs contributes DNA end ligation through the recruitment of x-ray cross-complementing gene 4 (XRCC4) and DNA ligase IV (LIG4)^27^. Together, we investigated NHEJ in the *EGFR*-mutant lines using NU7441, which inhibits DNA-PKcs activity^28^. In the presence of NU7441, the number of X-ray-induced γH2AX foci was comparable regardless of the *EGFR* mutation status, i.e., the additive effect of NU7441 on the increase in X-ray-induced γH2AX foci number was smaller in *EGFR*-mutant lines than in wild-type lines (Fig. 3a, Supplementary Table 3). If the mutant EGFRs function in homologous recombination, an alternative to NHEJ in DSB repair, the additive effect of NU7441 should not depend on the *EGFR* mutation status. Therefore, these data indicate that *EGFR*-mutant cells are defective in NHEJ.

Finally, we examined the repair of carbon-ion beam-induced DSB in *EGFR*-mutant and wild-type cells. No significant difference in the number of γH2AX foci was observed after irradiation between *EGFR*-mutant and wild-type lines (Fig. 3b, Supplementary Table 3). Meanwhile, in *EGFR* wild-type cells, the box plots of irradiation-alone and that of irradiation plus NU7441 were closer in carbon-ion beams than in X-rays. Furthermore, when the cells were irradiated with X-rays or carbon-ion beams for the same dose of 2 Gy, the number of γH2AX foci was significantly smaller in X-rays than in carbon-ion beams, in all the *EGFR*-mutant and the *EGFR* wild-type cells examined (Supplementary Fig. 2). Taken together, these data indicate that the repair efficacy for carbon-ion beam-induced DSBs is lower than that of X-ray-induced DSBs regardless of the *EGFR* mutation status. The low repair efficacy of carbon-ion beam-induced DSBs may be attributable in part to the structural complexity of the DSB ends^29^,^30^.

The results of the present study highlight the potential value of the *EGFR* mutation status as a predictor of CIRT RBE. To the best of our knowledge, this study is the first to report the association of driver gene mutation status with the RBE of carbon-ion beams. The results suggest that NSCLCs driven by mutations in oncogenes other than *EGFR*, including *KRAS*, show high RBE and thus should be selected as candidates for CIRT. Validation in animal models should be conducted. Nevertheless, previous research demonstrates an excellent agreement between the radiosensitivity of cancer cells assessed by clonogenic survival assay and the clinical response to radiotherapy^31^,^32^,^33^. Therefore, our results provide a valuable biological basis for selecting NSCLC patients for CIRT.

The present study focused on *EGFR* and *KRAS*, however, alterations in other genes were identified as drivers of NSCLC^13^,^14^. For example, H1299 cells carry a Q61K mutation in the oncogene *NRAS*^18^. Moreover, recent genome-wide analyses and functional validation demonstrated that genes that have not been recognized as classical oncogenic drivers, including genes involved in chromatin remodeling and DNA damage responses, are frequently mutated in human cancers, underscoring the pathogenic significance of these mutations^34^,^35^. Studies assessing the association between the mutation status of a wide panel of cancer-related genes and the sensitivity of cancer cells to X-rays and carbon-ion beams will further elucidate genetic profiles that affect radiosensitivity and RBE, and will provide biological basis for the establishment of useful predictors for personalized radiotherapy. To this end, the mutational analysis of 409 known cancer-related genes in the 15 NSCLC lines used in the present study is ongoing.

In summary, *EGFR* mutation-negative NSCLCs show a high RBE compared to *EGFR*-mutant NSCLCs and may, therefore, benefit from CIRT.

## Methods

### Cell lines

The human NSCLC lines A427, A549, H1299, H1650, H1703, H1975, H460, H520, H522, and HCC827 were purchased from ATCC (Manassas, VA, USA). LK2 and II-18 were purchased from JCRB Cell Bank. H157, Ma-24, and PC9 were provided by Dr. Harris (National Institute of Health), Dr. Shimizu (Tokushima University), and Dr. Kato (Tokyo Medical Collage), respectively. A549-WT, −ΔE746-A750, and −L858R cells were established as described previously^23^,^36^. All cell lines were cultured in RPMI-1640 (Sigma-Aldrich, St. Louis, MO, USA) supplemented with 10% fetal bovine serum (Life Technologies, Carlsbad, CA, USA).

### Irradiation

X-ray irradiation was performed using a Faxitron RX-650 (100 kVp, 1.14 Gy/min; Faxitron Bioptics, Tucson, AZ, USA). Carbon-ion beam irradiation was performed at the Gunma University Heavy Ion Medical Center using the same beam specifications used in clinical settings (290 MeV/nucleon and an average linear energy transfer at the center of a 6 cm spread-out Bragg peak of approximately 50 keV/μm). Carbon-ion beams were delivered in a vertical direction so that cells on culture plates could receive the dose evenly.

### Clonogenic survival assay

Cells were seeded into 6-well plates and exposed to X-rays or carbon-ion beams. After incubation for 10 days, the cells were fixed with methanol and stained with crystal violet. Colonies of at least 50 cells were counted. The surviving fraction was normalized to the corresponding controls. The D_10_ was calculated using the linear-quadratic model as described previously^37^.

### Immunofluorescence staining

Cells were seeded on glass coverslips in 35 mm dishes and incubated overnight. The culture medium was changed to that containing 10 μM of the DNA-PKcs inhibitor NU7441 (R&D Systems, Minneapolis, MN, USA). After incubation for 1 h, the cells were exposed to X-rays or carbon-ion beams. After incubation for 24 h, the coverslips were stained with antibodies against γH2AX (Merck Millipore, Billerica, MA, USA) as described previously^38^. The number of γH2AX foci per nucleus was scored in sequential 2D images captured from multiple focal planes using a fluorescence microscope (Eclipse Ni, Nikon, Tokyo, Japan) at ×100 magnification.

### Statistical analysis

Statistical analysis was performed using SigmaPlot 12.0 (Hulinks, Tokyo, Japan)^28^. Normality was tested by Shapiro-Wilk test. For the data sets that followed a normal distribution, variance was further tested by Levene Median test; if the variance was equal, significance was then tested by unpaired Student’s t-test. For the data sets that did not follow a normal distribution, significance was tested by Man-Whitney U test. *P* < 0.05 was considered significant.

## Additional Information

**How to cite this article**: Amornwichet, N. *et al.* The *EGFR* mutation status affects the relative biological effectiveness of carbon-ion beams in non-small cell lung carcinoma cells. *Sci. Rep.* **5**, 11305; doi: 10.1038/srep11305 (2015).

## Supplementary Material

Supplementary Information
