# Supplementary material for: The EGFR mutation status affects the relative biological effectiveness of carbon-ion beams in non-small cell lung carcinoma cells
Source: Sci Rep. 2015 Jun 11;5:11305. doi: 10.1038/srep11305 (PMC4463964; doi:10.1038/srep11305)
Supplement: Supplementary Information [file srep11305-s1.pdf]

Supplementary information

Title:

The *EGFR* mutation status affects the relative biological effectiveness of carbon-ion beams in non-small cell lung carcinoma cells

Authors:

Napapat Amornwichet[1,2], Takahiro Oike[1,3], Atsushi Shibata[4], Chaitanya S. Nirodi[5], Hideaki Ogiwara[3], Haruhiko Makino[6], Yuka Kimura[1], Yuka Hirota[1], Mayu Isono[7], Yukari Yoshida[7], Tatsuya Ohno[7], Takashi Kohno[3], Takashi Nakano[1]

Affiliation:

[1]Department of Radiation Oncology, Gunma University Graduate School of Medicine, Maebashi, Gunma, Japan; [2]Department of Radiology, Chulalongkorn University, Pathumwan, Bangkok, Thailand; [3]Division of Genome Biology, National Cancer Center Research Institute, Chuo-ku, Tokyo, Japan; [4]Advanced Scientific Research Leaders Development Unit, Gunma University, Maebashi, Gunma, Japan; [5]Department of Oncologic Sciences, Mitchell Cancer Institute, Alabama, USA; [6]Tottori University Hospital Cancer Center, Yonago, Tottori, Japan; [7]Gunma University Heavy Ion Medical Center, Maebashi, Gunma, Japan

Corresponding author:

Takahiro Oike, MD, PhD
Department of Radiation Oncology
Gunma University Graduate School of Medicine
3-39-22, Showa-machi, Maebashi, Gunma, 371-8511, Japan
Tel.: +81-283-22-5222; Fax: +81-283-22-8252
E-mail: oiketakahiro@gmail.com

Supplementary Figure 1

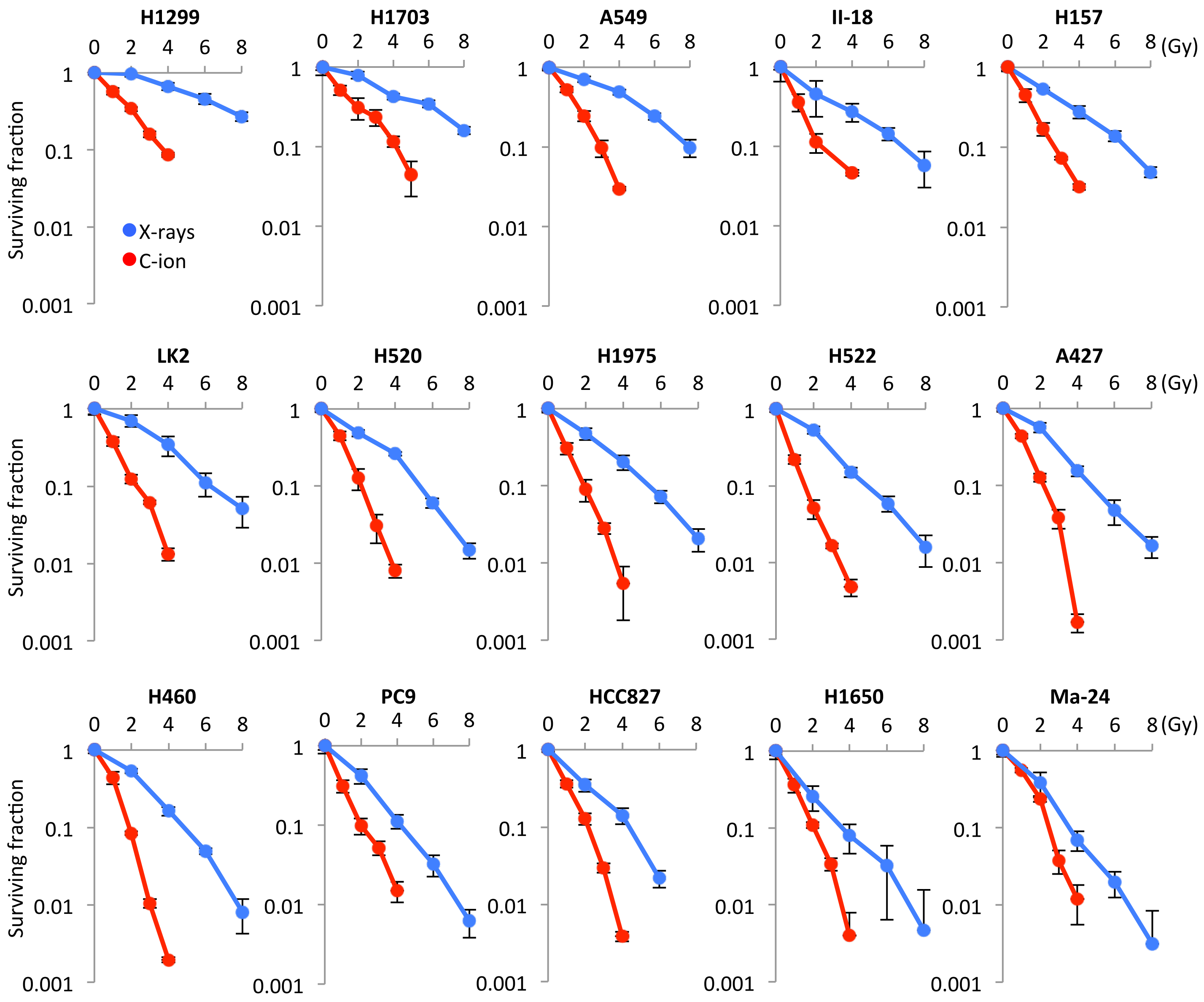

Supplementary Figure 1. Sensitivity of 15 NSCLC lines to X-rays or carbon-ion beams assessed by clonogenic survival assay. Data are represented as the mean ± SD. C-ion, carbon-ion.

Supplementary Figure 2

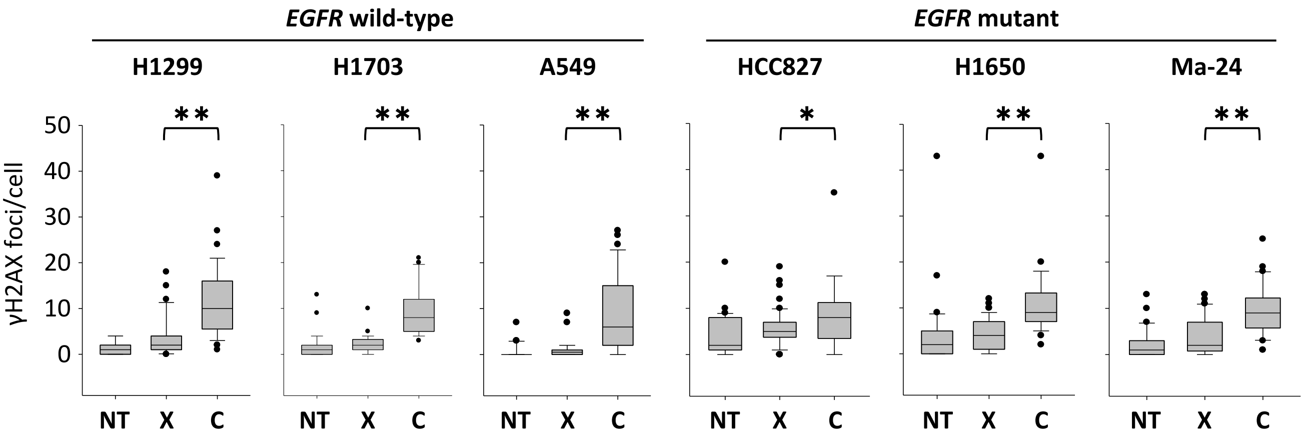

Supplementary Figure 2. Repair of X-ray- or carbon-ion beam-induced DSBs in *EGFR*-mutant or wild-type NSCLC lines assessed by immunofluorescence staining of γH2AX. Cells were exposed to X-rays (2 Gy) or carbon-ion beams (2 Gy) and stained with an antibody to γH2AX 24 h post-irradiation. The number of γH2AX foci per nucleus was scored in 30–50 cells for each experimental condition using a fluorescence microscope at ×100 magnification. The results of a representative experiment are shown as box plots. Note that the data of non-treated controls and those of carbon-ion beams are the same as in Figure 3 but now in the different context. ∗, $P < 0.05$; ∗∗, $P < 0.001$. NT, non-treated controls; X, X-rays; C, carbon-ion beams.

Supplementary Table 1

Supplementary Table 1. Mutation status in *EGFR* and *KRAS* in NSCLC lines

Cell line	Histopathology	*EGFR*	*KRAS*	Reference
Ma-24	Adenocarcinoma	L858R, E709G	Wild-type	15, 16
PC9	Adenocarcinoma	ΔE746_A750	Wild-type	15, 17
II-18	Adenocarcinoma	L858R	Wild-type	15, 17, 18
H1650	Adenocarcinoma	ΔE746_A750	Wild-type	15, 19
H1975	Adenocarcinoma	L858R, T790M	Wild-type	17, 19, 20
HCC827	Adenocarcinoma	ΔE746_A750	Wild-type	19, 20
A427	Adenocarcinoma	Wild-type	G12D	15, 18
A549	Adenocarcinoma	Wild-type	G12S	15, 18-20
H157	Squamous cell carcinoma	Wild-type	G12R	15, 18, 19
H460	Large cell carcinoma	Wild-type	Q61H	15, 18-20
H522	Adenocarcinoma	Wild-type	Wild-type	15
H1703	Adenocarcinoma	Wild-type	Wild-type	15, 19
H520	Squamous cell carcinoma	Wild-type	Wild-type	15, 19, 20
LK2	Squamous cell carcinoma	Wild-type	Wild-type	15, 17
H1299	Large cell carcinoma	Wild-type	Wild-type	15

Supplementary Table 2

Supplementary Table 2. D_{10} for X-rays and carbon-ion beams, and RBE in A549-WT, -ΔE746-A750 and -L858R cells

	D_{10} (X-rays)	D_{10} (C-ion)	RBE
A549-WT	7.0	3.7	1.9
A549-ΔE746-A750	5.6	3.6	1.5
A549-L858R	4.7	3.6	1.3

C-ion, Carbon-ion.

Supplementary Table 3

Suplementary Table 3. *P* values on the significant differences in the number of γH2AX foci

	X-ray alone						X-ray + NU7441						Carbon-ion beams alone						Carbon-ion beams + NU7441					
	H1299	H1703	A549	HCC827	H1650	Ma-24	H1299	H1703	A549	HCC827	H1650	Ma-24	H1299	H1703	A549	HCC827	H1650	Ma-24	H1299	H1703	A549	HCC827	H1650	Ma-24
H1299	-	0.68	0.0075	3.9E-07	1.4E-07	5.3E-07	-	0.38	0.41	0.53	0.76	0.06	-	0.88	0.73	0.46	0.38	0.99	-	0.38	0.41	0.53	0.76	0.056
H1703	-	-	0.0046	9.6E-07	4.8E-07	1.6E-06	-	-	0.11	0.16	0.27	0.0083	-	-	0.82	0.51	0.29	0.88	-	-	0.76	0.16	0.93	0.76
A549	-	-	-	8.4E-10	3.4E-10	2.8E-09	-	-	-	0.86	0.64	0.32	-	-	-	0.72	0.27	0.74	-	-	-	0.13	0.82	0.58
HCC827	-	-	-	-	0.53	0.62	-	-	-	-	0.77	0.25	-	-	-	-	0.14	0.45	-	-	-	-	0.28	0.27
H1650	-	-	-	-	-	0.91	-	-	-	-	-	0.15	-	-	-	-	-	0.35	-	-	-	-	-	0.75
